# CPR Compression Rotation Every One Minute Versus Two Minutes: A Randomized Cross-Over Manikin Study

**DOI:** 10.1155/2020/5479209

**Published:** 2020-09-01

**Authors:** Nutthapong Pechaksorn, Veerapong Vattanavanit

**Affiliations:** ^1^Division of Internal Medicine, Faculty of Medicine, Prince of Songkla University, Hat Yai, Songkhla 90110, Thailand; ^2^Critical Care Medicine Unit, Division of Internal Medicine, Faculty of Medicine, Prince of Songkla University, Hat Yai, Songkhla 90110, Thailand

## Abstract

**Background:**

The current basic life support guidelines recommend two-minute shifts for providing chest compressions when two rescuers are performing cardiopulmonary resuscitation. However, various studies have found that rescuer fatigue can occur within one minute, coupled with a decay in the quality of chest compressions. Our aim was to compare chest compression quality metrics and rescuer fatigue between alternating rescuers in performing one- and two-minute chest compressions.

**Methods:**

This prospective randomized cross-over study was conducted at Songklanagarind Hospital, Hat Yai, Songkhla, Thailand. We enrolled sixth-year medical students and residents and randomly grouped them into pairs to perform 8 minutes of chest compression, utilizing both the one-minute and two-minute scenarios on a manikin. The primary end points were chest compression depth and rate. The secondary end points included rescuers' fatigue, respiratory rate, and heart rate.

**Results:**

One hundred four participants were recruited. Compared with participants in the two-minute group, participants in the one-minute group had significantly higher mean (standard deviation, SD) compression depth (mm) (45.8 (7.2) vs. 44.5 (7.1), *P*=0.01) but there was no difference in the mean (SD) rate (compressions per min) (116.1 (12.5) vs. 117.8 (12.4), *P*=0.08), respectively. The rescuers in the one-minute group had significantly less fatigue (*P* < 0.001) and change in respiratory rate (*P* < 0.001), but there was no difference in the change of heart rate (*P*=0.59) between the two groups.

**Conclusion:**

There were a significantly higher compression depth and lower rescuer fatigue in the 1-minute chest compression group compared with the 2-minute group. This trial is registered with TCTR20170823001.

## 1. Introduction

Chest compression is the key component of cardiopulmonary resuscitation (CPR). The compression generates blood flow and oxygen delivery to the myocardium and brain by directly compressing the heart which increases intrathoracic pressure. Effective chest compression correlates with optimal coronary perfusion pressure and cardiac output resulting in a better chance for return of spontaneous circulation [1]. The latest basic life support guidelines from the American Heart Association recommends that rescuers should use a 2-minute chest compression rotation, or shorter if they feel fatigued, and the compression depth should be 5–6 cm with a rate of 100–120 per minute with minimal compression interruptions [2].

Various studies have found that the quality of chest compressions decreases dramatically after a short period. One report showed a significant reduction in chest compression performance from 79.7% in the first minute to 24.9% in the second [3]. Another report found that the percentage of correct chest compressions decreased significantly after 1 minute of compressions with a decline in adequate compression of 18.6% per minute [4].

Previous studies have been somewhat conflicting, with some studies finding a superior percentage of appropriate compression depth with lower fatigue when rescuers swapped every minute compared with every two minutes [5, 6]. Another study found no significant difference in the number of effective chest compressions between the two groups over the entire 8-minute scenario [7]. However, most studies [3, 4, 6–9] done before the 2015 guidelines concerning compression depth and rate were updated. In addition, some studies assessed chest compression quality only and did not assess rescuer fatigue or interruption times [3–5].

In the current study, we hypothesized that rotating the rescuers every minute would provide higher quality compressions and lower rescuer fatigue compared with a 2-minute rotation.

## 2. Methods

### 2.1. Study Design

This was a single center, prospective randomized cross-over study comparing the quality of chest compression and rescuers' fatigue between 1-minute and 2-minute rotations performed by trained sixth-year medical students and residents. The study design was approved by the Institutional Review Board (REC 60-123-14–4). All participants gave written informed consent before commencement of the study.

### 2.2. Patient and Public Involvement

No patients were involved.

### 2.3. Study Setting

The study was conducted in Songklanagarind Hospital using an adult cardiac arrest simulation. A Laerdal Resusci Anne^®^ (Laerdal Medical, Stavanger, Norway) training manikin was used as a simulated cardiac arrest patient. The participants assumed the role of a health care provider performing two-rescuer CPR. The CPR time was set at 8 minutes. Adequate compression depth was set between 50 and 60 mm and the adequate compression rate was set between 100 and 120 compressions per minute based on the Adult Basic Life Support 2015 American Heart Association Guidelines.

We recruited sixth-year medical students and residents of any specialty from the study hospital who had completed a basic life support course within the last 2 years. The period of recruitment was from September 2017 to December 2018. We excluded participants who had underlying medical problems which might cause them harm when performing CPR. The participants were classified into a two-person group by the enrollment order. The participant groups were marked as groups A and B randomly by drawing lots. Recruitment, enrollment, and randomization were carried out by the authors. Participants of group A performed a 1-minute chest compression rotation, rested for 30 minutes, and then performed a 2-minute chest compression rotation. Participants of group B performed the 2-minute chest compression rotation first, rested for 30 minutes, and then performed the 1-minute chest compression rotation in the same setting. The 8-minute cycle consisted of the two rescuers switching after every 1 or 2 minutes. A 30-minute washout period was used between rotations to reduce fatigue and changes in heart rates and respiratory rates of the participants prior to the second rotation. The bag valve mask was used as the ventilation method during the CPR compressions with chest compression-to-ventilation ratio set at 30 : 2 for both groups. The compression parameters were collected through feedback data via SimPad PLUS with Skill Reporter (Laerdal Medical), with the results of each group combined in the gathering from the performance of the two compressors in the 8-minute cycle. Heart rates were obtained by fingertip pulse oximetry (Yuwell YX302 Display, Medical Equipment). A nurse assistant measured the respiratory rates of the rescuers. A visual analog scale (VAS) was used to record the participants' fatigue, with scores ranging from 0 (no fatigue) to 10 (extreme fatigue) [10]. The participants were requested to mark on the scale of a horizontal 10 cm line anchored at each end by words to specify the level of intensity of his/her fatigue.

Age, gender, body mass index (BMI), history of performing CPR in real-life situations, time (in months) since the last basic life support training, and regular physical activity (defined as at least 30 minutes of exercise at least 3 times a week) were recorded.

The primary outcome of the study was the chest compression quality which was assessed based on depth and rate of compressions. The secondary outcomes were rescuer's fatigue assessment, respiratory rate, heart rate, interruption time, and the percentage of full-chest recoil during CPR. We also investigated the participant's characteristics that were related to the percentage of adequate compression depth.

### 2.4. Statistical Analysis

The sample size was calculated using the percentage of compressions of adequate depth as the primary outcome variable. In a previous study [5], the mean percentage of compressions of adequate depth in the 1-minute group was 76.2% ± 35.3 and in the 2-minute group was 54.3% ± 40.0. We set the two-sided significance level at 0.05 and the power of the test at 80%. The minimum number of participants was determined to be 47 in each group [11].

All statistical analyses were performed using *R* version 3.6.0 (R Foundation for Statistical Computing, Vienna, Austria). Continuous variables are presented using the mean with standard deviation (SD) for normally distributed data and median with interquartile range (IQR) otherwise. Categorical variables are presented using frequencies and percentages. Test of normality was performed using the Shapiro–Wilk test. Either paired Student's *t*-tests or Wilcoxon signed-rank tests were used for statistical comparisons between the two groups depending on the distribution of data. To reduce the type-I error rate associated with multiple hypothesis testing, we used a threshold of 0.01 for assessing statistical significance.

## 3. Results

One hundred four health care providers participated in this study. The response rate was 100% and there were no dropouts after randomization. The general characteristics of the participants are shown in Table 1. Slightly more than half (51%) were male. Most of them (80%) were 6-year medical students and 83% had a history of performing chest compressions in a real-life situation.

### 3.1. Quality of Chest Compression

Table 2 shows a comparison of CPR quality parameters between the two groups. The mean (SD) compression depth [mm] (45.8 (7.2) vs. 44.5 (7.1), *P*=0.01), median (IQR) percentage of adequate compression depth (21.5 (7.3, 51.8) vs. 19.5 (2.0, 42.3), *P*=0.004), and median (IQR) interruption time (seconds) (28.0 (18.0, 56.8) vs. 13 (9.0, 56.8), *P*=0.001) were all significantly higher in the 1-minute group compared to the 2-minute group.

### 3.2. Rescuers' Fatigue

**Figure 1:**
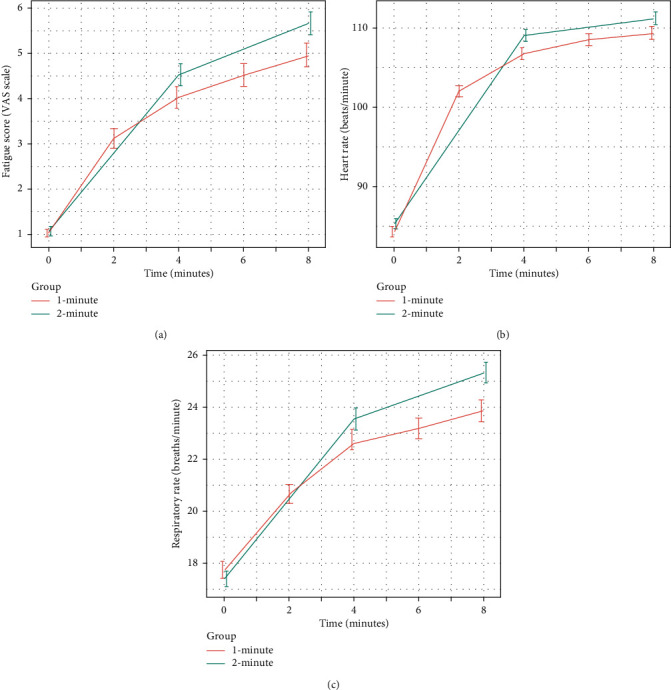
Change in rescuers' fatigability parameters in the 1- and 2-minute groups; mean fatigue score (a); mean heart rate (b); and mean respiratory rate (c).

Table 3 shows a comparison of fatigue indicators between the two groups. Participants in the 1-minute group had lower fatigue scores on the VAS (*P* < 0.001) and lower respiratory rate (*P* < 0.001) at the end of the 8-minute compression scenario compared to those in the 2-minute group; however, there was no difference in the change in heart rate between the two groups (*P*=0.59). Figures 1(a)–1(c) show that there was an upward trend in fatigue score, heart rate, and respiratory rate in both groups after each rotation, but the increase was less dramatic for participants in the 1-minute group.

### 3.3. Factors Associated with Achieving Adequate Chest Compression

Factors associated with differences between the two study groups in achieving adequate compression depth between various subgroups are presented in Table 4. Among males, those with a BMI less than 25 kg/m^2^, with no history of CPR in a real-life situation, whose last CPR training session was within the past 6 months, and those who did regular physical activity, the percentage of adequate compression depth was significantly higher in the 1-minute group.

## 4. Discussion

The study found a significantly higher mean compression depth and lower rescuer fatigue and respiratory rate after 8 minutes among the 1-minute chest compression group compared with the 2-minute group.

These results are similar to a previous study by Gianotto-Oliveira et al. which found a significantly higher compression depth (76.21 vs. 54.34, *P* < 0.001) and percentage of adequate compression depth (76.2% vs. 54.3%, *P* < 0.001) and significantly lower fatigue (1.99 vs 4.56, *P* < 0.001) in their 1-minute group compared to their 2-minute group [5]. However, most of the participants in that study were male (85%) which may have affected their results. In our study, which had a similar proportion of males and females, we found significantly higher rates of adequate compression depth and lower rescuer fatigue.

Another cross-over study which compared 1-minute and 2-minute groups over the entire 8-minute scenario found no significant differences in the number of effective chest compressions between the two groups (mean number of effective chest compressions in the 1-minute group was 573.4 versus 597.6 in the 2-minute group) [7]. However, a compression depth of 38 mm or greater, following the old guideline [2], was used to define adequacy. A recent cross-over study [12] compared 1-minute and 2-minute continuous chest compressions, in terms of chest compression only CPR quality metrics on a manikin model over a four-minute time period, and found no statistically significant difference in the percentage of adequate compression depth (42.9% vs. 39.6%, *P*=0.66). However, the compression duration of 4 minutes might not have been long enough to achieve statistical significance.

In our study, we found a significantly higher total interruption time in the 1-minute group compared to the 2-minute group (28 vs. 13 sec, *P*=0.01) during the 8-minute compression period. When assuming the appropriate rate of 100 compressions per minute, the approximate interruption time was 5.8 compressions per minute in the 1-minute group and 2.7 compressions per minute in the 2-minute group. The chest compression fraction is a measurement of the proportion of CPR time performing chest compression. According to the latest guidelines [2], the chest compression fraction should be as high as possible, with a target of at least 60%. The benefit of shorter uninterrupted chest compressions on coronary perfusion pressure was demonstrated in a swine model by Ewy et al. [13]. In our study, the chest compression fractions were 94% and 97% in the 1-minute and 2-minute groups, respectively, both of which are acceptable in clinical practice. However, the value of the percentage of chest compression fraction in our study was quite high [14, 15]. The possible reasons may lie in the simulation environment, as rescuers were not interfered with compared to the real situation, and the time used for pulse checking and ventilation was shorter than in real practice.

The compression depth and overall percentage of adequate compression depth in our study were quite low compared to other studies [5, 7, 12] and the most recent recommended guideline [2]. There are two reasons for these results. First, due to this being a manikin study, the participants may not do the chest compression intentionally compared with a real-life situation. Second, we did not evaluate basic life support performance of all participants before enrollment.

Only one-fifth of compressions were of adequate depth in both groups. There was a statistically significant difference in favor of 1-minute cycles but a small real-world difference (19.5% vs. 21.5%). The number of interruptions to CPR was less with 2-minute cycles and the rate of compression was better in the 2-minute cycles although the difference was not statistically significant. Rescuers therefore should consider rotating their roles every minute to improve the quality of CPR and lower fatigue.

The factors related to higher rates of adequate compression depth were male and recent (<6 months) CPR training. In general, males have a better physical build and strength than females. Although it is a subject already addressed in the literature, this article brings different points of view, according to the latest resuscitation guidelines that can contribute to new studies about CPR. In terms of time since last CPR training, a previous study reported that core skills and knowledge decay within 3 to 12 months after initial CPR training [16]. The study emphasized the importance of regular training refreshers for improving the quality of CPR.

Despite the elimination of many confounding factors via randomization and the use of a cross-over design where participants acted as their own controls, this study had some limitations. First, this was a manikin study, and manikins are not a perfect substitute for the real human body. A simulated situation will not have the potential psychogenic stresses which can have an impact on personal efforts. Second, our study was conducted in a single center. Third, the participants in our study were medical students and residents; thus results may not represent the abilities of other health professional groups such as nurses or senior doctors. Fourth, results of the secondary outcome and correlation between the selected variables and the percentages of adequate chest compression depth should be interpreted with caution because of the small sample size. Finally, we collected data only for 8 minutes, a duration that is suitable for investigation purposes. Studies investigating compression times with longer durations may give different results.

## 5. Conclusion

This study found that the performance of 1-minute alternating compressions gave a higher number of adequate compressions and lower rescuer fatigue compared with a 2-minute compressions cycle. We suggest that in real-life situations, rescuers should consider rotating their roles every minute to improve the quality of CPR and reduce their fatigue.

## Figures and Tables

**Table 1:** Baseline characteristics of the study participants (*N* = 104).

Characteristic	Value
Male, *n* (%)	53 (51.0)
Age (years)	24 (23, 24)
BMI (kg/m^2^)	21 (20, 24)
Stage of medical training, *n* (%)	
6-year student	83 (79.8)
Postgraduate (first year)	11 (10.6)
Postgraduate (second year)	10 (9.6)
History of performing CPR in real-life situations, *n* (%)	86 (82.7)
Last CPR training session (months)	7 (4, 11)
Regular physical activity, *n* (%)	24 (23.1)

Data are presented as median (interquartile range) or *n* (%); BMI, body mass index; CPR, cardiopulmonary resuscitation.

**Table 2:** Comparison of cardiopulmonary resuscitation quality parameters between the two groups.

Parameter	1-minute group	2-minute group	*P* value
Chest compression rate (per min)	116.1 ± 12.5	117.8 ± 12.4	0.077
Chest compression depth (mm)	45.8 ± 7.2	44.5 ± 7.1	0.010
Adequate compression depth (%)	21.5 (7.3, 51.8)	19.5 (2.0, 42.3)	0.004
Adequate compression rate (%)	36.0 (20.5, 74.5)	51.5 (28.0, 83.8)	0.163
Interruption time (seconds)	28.0 (18.0, 56.8)	13 (9.0, 56.8)	0.001
Correct hand position (%)	100 (85.0, 100)	100 (76.0, 100)	0.509
Full-chest recoil (%)	53.9 ± 29.6	51.1 ± 31.2	0.162

Data are presented as mean ± standard deviation or median (interquartile range).

**Table 3:** Comparison of rescuer's fatigability parameters (heart rate, respiratory rate, and fatigue) at baseline and at the end of each rotation between the two groups.

Parameter	1-minute group	2-minute group	*P* value
Heart rate (beats/min)			
Baseline Final	84.36 ± 12.03 109.36 ± 17.29	85.30 ± 11.49 111.19 ± 18.02	0.360 0.218
Change	25.00 ± 15.43	25.9 ± 15.42	0.596
Respiratory rate (breaths/min)			
Baseline Final	17.73 ± 2.65 23.85 ± 4.48	17.40 ± 2.32 25.32 ± 4.20	0.122 < 0.001
Change	6.13 ± 4.71	7.91 ± 4.40	<0.001
Fatigue (VAS score)			
Baseline	1.03 ± 0.17	1.07 ± 0.25	0.071
Final	4.96 ± 1.76	5.66 ± 1.64	<0.001
Change	3.93 ± 1.78	4.60 ± 1.66	<0.001

Data are presented as mean ± standard deviation; VAS: visual analogue scale.

**Table 4:** Comparison of adequate chest compression depth percentage between various subgroups.

Variable	Group		*P* value^a^
	1-minute	2-minute	
Gender			
Male (*n* = 53)	36.4 (9.7, 82.8)	11.9 (1.7, 60.4)	<0.001
Female (*n* = 51)	1.7 (0, 26.3)	0.6 (0, 10.0)	0.010
Body mass index			
<25 kg/m^2^ (*n* = 83)	12.2 (0, 58.4)	4.5 (0, 24.0)	<0.001
≥25 kg/m^2^ (*n* = 21)	21.1 (3.2, 85.6)	11.6 (0.5, 92.8)	0.150
History of CPR in a real-life situation, *n* (%)			
Yes (*n* = 86)	14.0 (0, 51.5)	4.2 (0, 28.2)	0.130
No (*n* = 18)	35.0 (2, 83.3)	11.3 (1, 49.5)	<0.001
Last CPR training session			
<6 months ago (*n* = 31)	33.5 (7.1, 67.3)	20.2 (2.8, 59.3)	0.010
≥6 months ago (*n* = 73)	9.7 (0, 46.9)	2.7 (0, 16.0)	<0.001
Regular physical activity, *n* (%)			
Yes (*n* = 24)	49.7 (12.8, 81.0)	9.9 (1.9, 53.5)	0.020
No (*n* = 80)	8.7 (0, 39.7)	4.1 (0, 23.8)	<0.001

Data are presented as median (interquartile range); CPR, cardiopulmonary resuscitation. ^a^Between groups.

## Data Availability

The data from this study are available from the corresponding author upon request.

## References

[1] Lurie K. G., Nemergut E. C., Yannopoulos D., Sweeney M. (2016). The physiology of cardiopulmonary resuscitation. *Anesthesia & Analgesia*.

[2] Kleinman M. E., Brennan E. E., Goldberger Z. D. (2015). Part 5: adult basic life support and cardiopulmonary resuscitation quality. *Circulation*.

[3] Ochoa F. J., Ramalle-Gómara E., Lisa V., Saralegui I. (1998). The effect of rescuer fatigue on the quality of chest compressions. *Resuscitation*.

[4] Hightower D., Thomas S. H., Stone C. K., Dunn K., March J. A. (1995). Decay in quality of closed-chest compressions over time. *Annals of Emergency Medicine*.

[5] Gianotto-Oliveira R., Gianotto-Oliveira G., Gonzalez M. (2015). Quality of continuous chest compressions performed for one or two minutes. *Clinics*.

[6] Huseyin T. S., Matthews A. J., Wills P., O’Neill V. M. (2002). Improving the effectiveness of continuous closed chest compressions: an exploratory study. *Resuscitation*.

[7] Manders S., Geijsel F. E. C. (2009). Alternating providers during continuous chest compressions for cardiac arrest: every minute or every two minutes?. *Resuscitation*.

[8] Ashton A., McCluskey A., Gwinnutt C. L., Keenan A. M. (2002). Effect of rescuer fatigue on performance of continuous external chest compressions over 3 min. *Resuscitation*.

[9] Bjørshol C. A., Søreide E., Torsteinbø T. H., Lexow K., Nilsen O. B., Sunde K. (2008). Quality of chest compressions during 10min of single-rescuer basic life support with different compression: ventilation ratios in a manikin model. *Resuscitation*.

[10] Riera S. Q., González B. S., Álvarez J. T., Fernández M. D. M. F., Saura J. M. (2007). The physiological effect on rescuers of doing 2min of uninterrupted chest compressions. *Resuscitation*.

[11] Ngamjarus C., Chongsuvivatwong V. (2014). n4Studies: sample size and power calculations for iOS.

[12] Kilic D., Goksu E., Kilic T., Buyurgan C. S. (2018). Resuscitation quality of rotating chest compression providers at one-minute vs. two-minute intervals: a mannequin study. *American Journal of Emergency Medicine*.

[13] Ewy G. A., Zuercher M., Hilwig R. W. (2007). Improved neurological outcome with continuous chest compressions compared with 30:2 compressions-to-ventilations cardiopulmonary resuscitation in a realistic swine model of out-of-hospital cardiac arrest. *Circulation*.

[14] Christenson J., Andrusiek D., Everson-Stewart S. (2009). Chest compression fraction determines survival in patients with out-of-hospital ventricular fibrillation. *Circulation*.

[15] Iyanaga M., Gray R., Stephens S. W. (2012). Comparison of methods for the determination of cardiopulmonary resuscitation chest compression fraction. *Resuscitation*.

[16] Bhanji F., Mancini M. E., Sinz E. (2010). Part 16: education, implementation, and teams: 2010 American heart association guidelines for cardiopulmonary resuscitation and emergency cardiovascular care. *Circulation*.

